# Erchen Decoction Prevents High-Fat Diet Induced Metabolic Disorders in C57BL/6 Mice

**DOI:** 10.1155/2015/501272

**Published:** 2015-10-04

**Authors:** Bi-Zhen Gao, Ji-Cheng Chen, Ling-Hong Liao, Jia-Qi Xu, Xiao-Feng Lin, Shan-Shan Ding

**Affiliations:** ^1^Fujian University of Traditional Chinese Medicine, Fujian 350122, China; ^2^Fujian Key Laboratory of TCM Health State, Fujian University of Traditional Chinese Medicine, Fujian 350122, China; ^3^Department of Endocrinology, Quanzhou City Hospital of Traditional Chinese Medicine, Fujian 362002, China

## Abstract

Erchen decoction (ECD) is a traditional Chinese medicine prescription, which is used in the treatment of obesity, hyperlipidemia, fatty liver, diabetes, hypertension, and other diseases caused by retention of phlegm dampness. In this study we investigated the potential mechanism of ECD, using metabolism-disabled mice induced by high-fat diet. Body weight and abdominal circumference were detected. OGTT was measured by means of collecting blood samples from the tail vein. Blood lipid levels and insulin were measured using biochemical assay kit. Real-time PCR was used to measure the CDKAL1 gene expression and western blot was used to measure the protein expression. Through the research, it was found that ECD showed markedly lower body weight and abdominal circumference than those in the HFD group. Consistently, we observed that ECD significantly improved glucose tolerance, promoted the secretion of insulin and decreased the level of TG, TC level. Meanwhile, we observed significantly increased CDKAL1 mRNA and protein level in the ECD group. Therefore, we speculate that the potential molecular mechanism of ECD is to promote the CDKAL1 expression, ameliorate islet cell function, and raise insulin levels to regulate the metabolic disorder.

## 1. Introduction

Erchen decoction (ECD) is a common Traditional Chinese Medicine prescription, which was first recorded in a classic clinical Traditional Chinese Medicine (TCM) book titled The Taiping Huimin Heji Jufang, and it is a basic prescription of the treatment in drying dampness and resolving phlegm and is used in the treatment of a variety of diseases caused by retention of phlegm dampness. In the clinical application of TCM, ECD is used extensively in the treatment of obesity, hyperlipidemia, fatty liver, diabetes, hypertension, and other diseases caused by retention of phlegm dampness [1–4]. It has been reported that the phlegm dampness tends to be concentrated in the metabolic syndrome people [5, 6]. At present, metabolic syndrome (MS) refers to the clinical syndrome gathered by diabetes, hyperlipidemia, and hypertension, which takes the insulin resistance as the basis and central obesity as the main performance. In the past decades, MS has attracted attention due to its higher and higher incidence related to changes of people's life style in China. It is shown in studies that ECD [7, 8] could improve the fatty deposits, reduce blood lipid, lower body mass index and blood glucose, improve the glycolipid metabolic disorder, and so on. It is found in animal experiments that ECD could reduce the blood glucose and blood lipid in mice fed with high-fat diet and increase insulin sensitivity [9, 10]. It also has the function of reducing the atherosclerosis index and antiatherosclerosis [11]. Despite multiple treatment effects observed, the molecular mechanism of ECD in regulating metabolism disorder is still unclear.

CDKAL1 is located on chromosome 6, 6P22.3, and its full length is 37 kb. The mRNA of CDKAL1 is highly expressed in the bone, muscle, pancreas, and brain tissues in the human body. In recent years, part of the study shows that CDKAL1 is a mammalian methylthiotransferase that catalyzes the 2-methylthio (ms2) modification of N6-threonylcarbamoyladenosine (t6A) to produce 2-methylthio-N6-threonylcarbamoyladenosine (ms2t6A) at position 37 of tRNALys (UUU) [12]. The ms2 modification of tRNALys (UUU) stabilises the interaction with its cognate codons, allowing for efficient translation. CDKAL1 induces insulin secretion through induction of precise protein translation of insulin [13]. Clinical studies and animal studies have suggested that ECD can promote the secretion of insulin. In this study, we would like to explore the molecular mechanisms of ECD in treatment of MS by observing its effect on the expression of CDKAL1 in mice fed with high-fat diet.

## 2. Materials and Methods

### 2.1. Drug Preparation and Diet

ECD comprises four Chinese herbs:* Pericarpium Citri Reticulatae* (9 g),* Rhizoma Pinelliae* (9 g),* Poria* (6 g), and* Radix Glycyrrhizae* (3 g). The dosage is determined according to the book of The Taiping Huimin Heji Jufang. All herbs were purchased from Guoyi Hospital affiliated to Fujian University of TCM. Herbal decoction was prepared in accordance with conventional TCM decocting methods. (1) Place all herbs in a cooking pot (porcelain) with 500 mL water; (2) boil the herbs with highest heat after 30 minutes of soak; (3) reduce heat and simmer for 20 minutes; (4) transfer the liquid by filtration; (5) add water and boil the remaining, and then repeat (3) and (4) one more time to make a second dose of medicine; and (6) mix the two doses in a glass pot and concentrate solution by the rotary evaporation apparatus. The final concentrated decoction is 50 mL. ECD, metformin, and simvastatin were administered at a dose of 10 mL/kg/d (pure solution), which was approximately 12 times of the standard dose in practice, according to the dose-equivalence equation between mice and humans [14]. The high-fat diet (HFD) contained 34.9% fat (60% of calories), 26.3% carbohydrates (20% of calories), and 26.2% protein (20% of calories) as well as fiber, vitamins, and minerals with total calorific value 21924 kJ/kg (D12492, Research Diets, New Brunswick, NJ, USA). The ordinary diet (NFD) contained 5% fat, 23% protein, and 53% carbohydrate with total calorific value 25 kJ/kg (Shanghai Slac Laboratory Animal Company, Shanghai, China).

### 2.2. Animals and Interventions

SPF animals (male C57BL/6J mice, 20 g ± 2 g) were obtained from Shanghai Slac Laboratory Animal Company (Shanghai, China). Mice were housed in an SPF, temperature (24°C ± 2°C) and humidity controlled (55% ± 10%) room with a 12-hour light-dark cycle (commencing with light at 08:00) in the animal experiment center of Fujian University of TCM. The experimental protocol was approved by the Fujian University of TCM Ethics Committee for the use of experimental animals (number 2014-004). Animals were randomized into 2 groups: the normal group (NFD, *n* = 8), fed ordinary diet, and the high-fat diet group (HFD, *n* = 32), fed high-fat diet. After 10 weeks, the mice in the HFD group were randomized into 4 groups: the model group (HFD, *n* = 8), the Erchen decoction group (ECD, *n* = 8), the metformin group (MFN, *n* = 8, each mouse is given 0.3 g/kg metformin), and the simvastatin group (SVN, *n* = 8, each mouse is given 2 mg/kg simvastatin). Every group, respectively, gives corresponding drug by gavage orally for 4 weeks. All mice could eat and drink ad libitum. Body weight and abdominal circumference were recorded every 2 weeks.

### 2.3. Intraperitoneal Glucose Tolerance Test

At week 10, week 12, and the end of the treatment, mice were fasted overnight (12 h). The baseline glucose values (0 min), prior to injection of glucose (1 g/kg body weight), were measured by means of collecting blood samples from the tail vein. Additional blood samples were collected at regular intervals (30, 60, and 120 min) for glucose tolerance tests.

### 2.4. Serum Chemistry Analysis

The mice were fasted overnight and anesthetized. Blood samples were collected from the retroorbital sinuses of each group. Serum triglyceride (TG), total cholesterol (TC), HDL cholesterol (HDL-c), and LDL cholesterol (LDL-c) were measured using biochemical assay kit according to the manufacturer's instruction (Nanjing Jiancheng Bioengineering Institute, Nanjing, China). Serum insulin was measured using an ELISA kit according to the manufacturer's instruction (Shanghai Westang Bio-Tech Co. Ltd., Shanghai, China).

### 2.5. Real-Time PCR for mRNA Analysis

Total RNA was extracted from liver subcutaneous adipose and visceral adipose tissues using Trizol reagent (TaKaRa, Otsu, Japan). First-strand complementary DNA (cDNA) was generated by reverse transcriptase, with random primers (TaKaRa, Otsu, Japan). The sequences of the primers are described in Table 1. To evaluate the mRNA expression of CDKAL1 in the liver subcutaneous adipose and visceral adipose tissues, real-time PCR was performed using a SYBR Green master mix kit (TaKaRa, Otsu, Japan) according to the manufacturer's instructions on the Mastercycler ep realplex4S real-time PCR system (Eppendorf, Hamburg, Germany). The cDNA was denatured at 95°C for 10 min followed by 40 cycles of PCR (95°C, 15 s; 60°C, 60 s). The 2^−ΔΔCt^ method [15] was used to determine relative amounts of product, and data are presented as fold change, using *β*-actin as an endogenous control.

### 2.6. Protein Isolation and Western Blotting

The liver subcutaneous adipose and visceral adipose tissues of every group were homogenized in liquid nitrogen, and whole-cell protein was extracted by using lysate buffer containing proteinase inhibitor (Beyotime Biotechnology, Shanghai, China). Protein concentration was quantified spectrophotometrically by using BSA protein assay kit (Beyotime Biotechnology, Shanghai, China). Protein samples were separated by PAGE using 10% SDS-polyacrylamide gels. Samples were transferred to polyvinylidene fluoride membrane and blocked with 5% milk. The membrane was incubated with a rabbit anti-CDKAL1 primary antibody (1 : 500, Abcam, Cambridge, England) overnight at 4°C and followed by the secondary antibody (against rabbit, Beyotime Biotechnology, Shanghai, China) for 1 h at 37°C. The primary antibodies including mouse anti-*β*-actin (1 : 1000, Sigma, America) were similar. Lastly, each protein band was detected using enhanced chemiluminescence (ECL, Beyotime Biotechnology, Shanghai, China). The densitometric values were measured with Gel-Pro Analyzer.

### 2.7. Statistical Analysis

Data analyses were performed using the statistical program SPSS 19.0. All data were presented as means ± SE. Independent-samples *t*-test was performed to compare two groups. ANOVA was performed to compare multiple groups. Differences were considered as significant, *P* < 0.05, or not significant, *P* > 0.05.

## 3. Results

### 3.1. ECD Reduces the Body Weight and Abdominal Circumference in Mice Fed with High-Fat Diet

**Figure 1:**
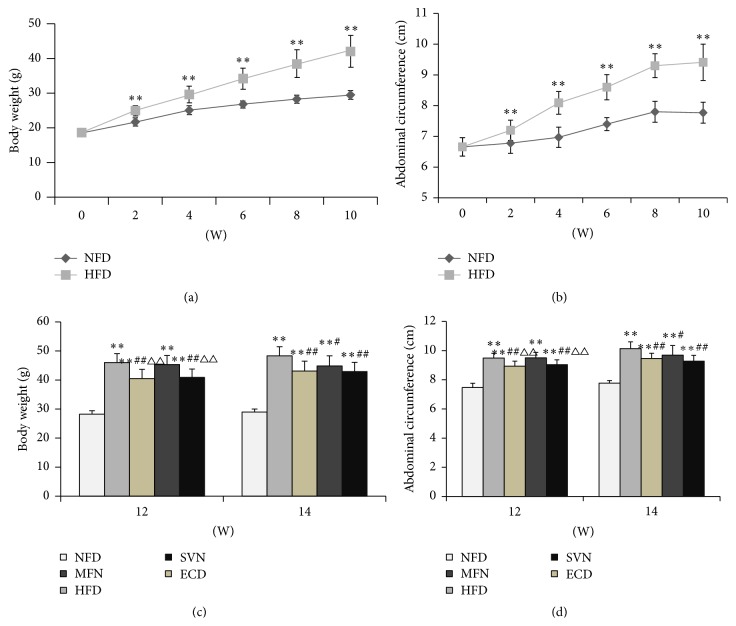
The changes of body weight and abdominal circumference: (a) body weight (weeks 0–10); (b) abdominal circumference (weeks 0–10); (c) body weight (weeks 12 and 14); and (d) abdominal circumference (weeks 12 and 14); ^*∗∗*^ *P* < 0.01, versus the NFD group; ^#^ *P* < 0.05, ^##^ *P* < 0.01, versus the HFD group; ^△△^ *P* < 0.01, versus the MFN group.

In order to explore ECD effects on body weight and abdominal circumference in high-fat mice, body weight and abdominal circumference were recorded every 2 weeks. Since the second week, the HFD group was significantly higher than the NFD group regarding body weight and abdominal circumference (*P* < 0.01) (Figures 1(a) and 1(b)). After the medication intervention, the body weight and abdominal circumference in the ECD and SVN groups were markedly lower than in the HFD group (*P* < 0.01) which also lost more weight and abdominal circumference than the MFN group at week 12 (*P* < 0.01). In addition, the body weight and abdominal circumference in the MFN group were significantly lower than in the HFD group at week 14 (*P* < 0.05) (Figures 1(c) and 1(d)).

### 3.2. ECD Improves the Glucose Tolerance in Mice Fed with High-Fat Diet

**Figure 2:**
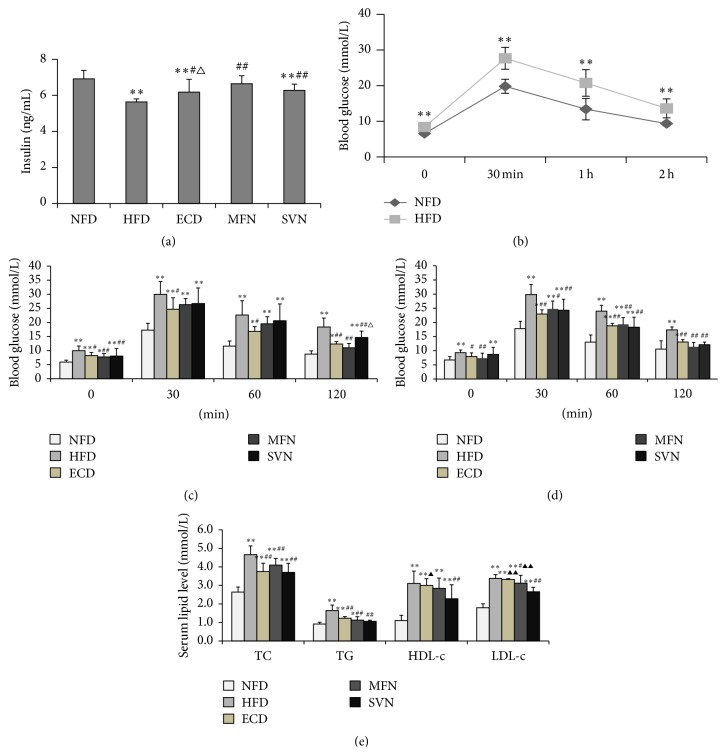
ECD improve insulin level, OGTT, and lipid accumulation: (a) insulin level; (b) OGTT (week 10); (c) OGTT (week 12); (d) OGTT (week 14); and (e) lipid levels; ^*∗*^ *P* < 0.05, ^*∗∗*^ *P* < 0.01, versus the NFD group; ^#^ *P* < 0.05, ^##^ *P* < 0.01, versus the HFD group; ^△^ *P* < 0.05, versus the MFN group; ^▲^ *P* < 0.05, ^▲▲^ *P* < 0.01, versus the SVN group.

We used the glucose tolerance test to explore the change of blood glucose. After high-fat feeding, the glucose tolerance was markedly lower in the HFD group (*P* < 0.01) (Figure 2(b)). After the medication intervention, fasting blood glucose significantly decreased in the ECD and MFN groups compared to the HFD group (*P* < 0.01); meanwhile, there was no significant difference between the ECD, MFN, and NFD groups at week 14 (*P* > 0.05). For the glucose tolerance, compared with the HFD group, the ECD group was significantly improved at week 12 and week 14 (*P* < 0.05), and the MFN and SVN groups were significantly improved at week 14 (*P* < 0.05) (Figures 2(c) and 2(d)).

### 3.3. ECD Improves the Level of Insulin and Reduces Blood Lipid Levels

We surmised that the ECD can possibly improve the glucose tolerance by changing the insulin levels. Later on, the study found that the insulin levels of the ECD, MFN, and SVN groups were significantly higher than the HFD group (*P* < 0.05), and the effect of MFN was better than that of ECD; the difference was statistically significant (*P* < 0.05) (Figure 2(a)). Compared with the NFD group, TC, TG, HDL-c, and LDL-c in the HFD group were higher, and the difference was statistically significant (*P* < 0.01); TC and TG in the ECD, MFN, and SVN groups decreased significantly compared to the HFD group (*P* < 0.01); LDL-c of the MFN and SVN groups decreased significantly compared to the HFD group (*P* < 0.05); HDL-c of the SVN group decreased significantly compared to the HFD group (*P* < 0.01). In addition, the SVN group was markedly lower than the ECD group regarding the level of HDL-c and LDL-c (*P* < 0.05). Meanwhile, compared with the MFN group, the level of LDL-c in the SVN group was significantly lower (*P* < 0.01) (Figure 2(e)).

### 3.4. ECD Increased the Expression of CDKAL1 in the Liver Visceral Adipose and Subcutaneous Adipose Tissues

**Figure 3:**
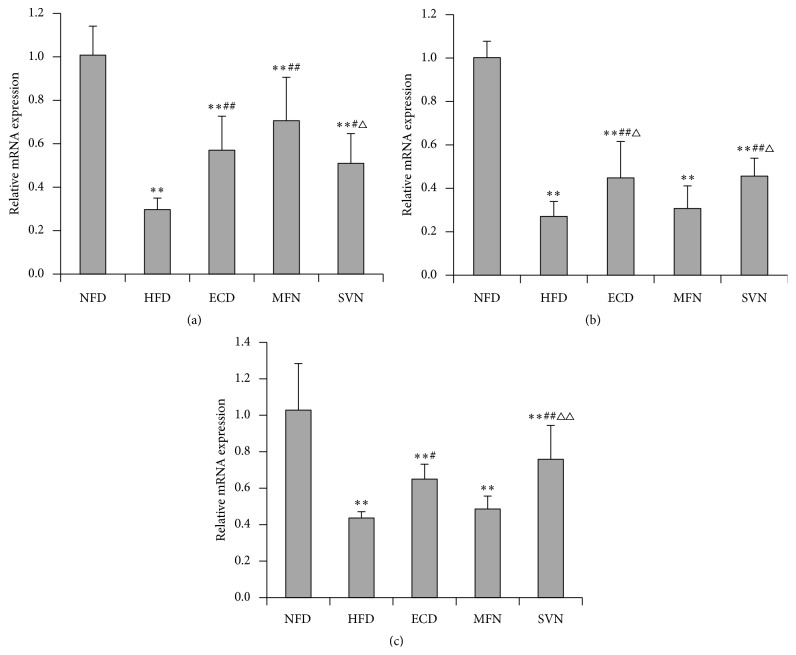
The mRNA expression of CDKAL1: (a) liver tissue; (b) visceral adipose tissue; and (c) subcutaneous adipose tissue; ^*∗∗*^ *P* < 0.01, versus the NFD group; ^#^ *P* < 0.05, ^##^ *P* < 0.01, versus the HFD group; ^△^ *P* < 0.05, ^△△^ *P* < 0.01, versus the MFN group.

**Figure 4:**
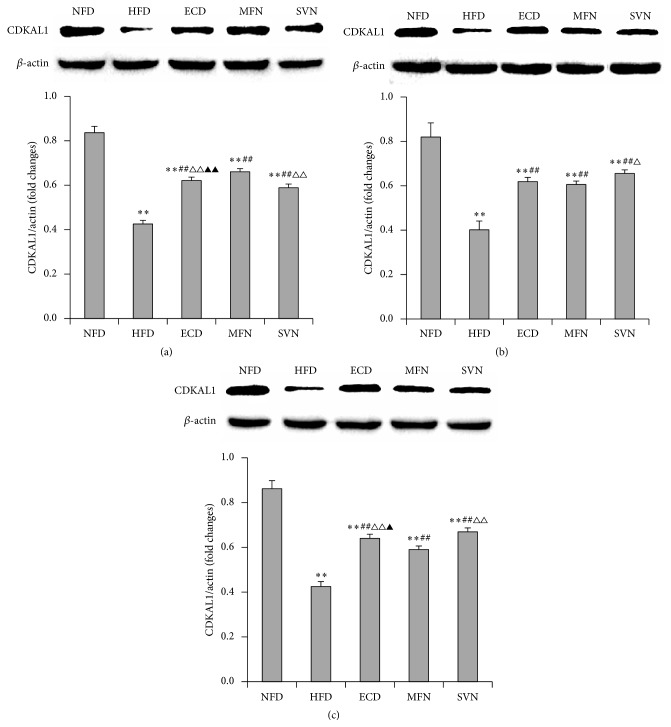
The protein expression of CDKAL1: (a) liver tissue; (b) visceral adipose tissue; and (c) subcutaneous adipose tissue; ^*∗∗*^ *P* < 0.01, versus the NFD group; ^##^ *P* < 0.01, versus the HFD group; ^△^ *P* < 0.05, ^△△^ *P* < 0.01, versus the MFN group; ^▲^ *P* < 0.05, ^▲▲^ *P* < 0.01, versus the SVN group.

To explore the mechanisms by which ECD regulate the metabolic disorder in high-fat mice, the expressions of CDKAL1 mRNA and protein were tested. The study found that the expression of CDKAL1 mRNA and protein in the HFD group decreased significantly in the liver, visceral adipose and subcutaneous adipose tissues (*P* < 0.01). In the liver tissues, the expressions of CDKAL1 mRNA and protein in the ECD, MFN, and SVN groups were significantly higher than that of the HFD group (*P* < 0.05); meanwhile, the MFN group was markedly higher than the SVN group (*P* < 0.05). Indeed, the MFN group was significantly higher than the ECD group regarding the expression of protein (*P* < 0.01) (Figures 3(a) and 4(a)). In visceral adipose and subcutaneous adipose tissue, compared with HFD group, the expression of mRNA and protein significantly elevated in the ECD group (*P* < 0.01), and the expression of protein significantly elevated in the MFN group (*P* < 0.01). In addition, the ECD group was markedly higher than the MFN group regarding the expression of mRNA (*P* < 0.05). Compared with the HFD and MFN groups, the expression of mRNA and protein markedly elevated in the SVN group (*P* < 0.05); meanwhile, the SVN group was significantly higher than the ECD group regarding the expression of protein (*P* < 0.05) (Figures 3(b), 3(c), 4(b), and 4(c)).

## 4. Discussion

The retention of phlegm dampness is considered to be an important factor in the metabolic disorders related diseases in the TCM theory. And according to the TCM theory, the high-fat diet causes the spleen and stomach impairment and then leads to the accumulation of grease in the body, and finally the excess grease induces the retention of phlegm dampness [16]. We fed the mice with the high-fat diet; after 2 weeks, the body weights and abdominal circumference of the mice increased significantly and the obvious abdominal obesity appeared. At the same time, the mice had significantly elevated blood glucose, impaired glucose tolerance, and lipid metabolism disorders. All of these symptoms are consistent with the pathological diagnosis of metabolic syndrome. Therefore, it is considered that the mice fed with high-fat diet can induce the model of the retention of phlegm dampness in MS.

A series of TCM studies have indicated that [5, 6] the retention of phlegm dampness is one of main TCM pathological factors of MS, which runs through the whole disease development process. As the basic prescription of the treatment in drying dampness and resolving phlegm, ECD showed a good effect in the treatment of MS. It was found in the animal experiments [17, 18] that ECD could reduce blood glucose, regulate lipid metabolism, and reduce the expression of IR, IRS-1, and Cav-1, so as to improve insulin resistance. Also ECD can decrease the NF-*κ*B excessive activation and inhibit the adipose tissue low-grade inflammation in MS model of rats [19]. Another study carried out in rats fed with HFD also showed that ECD can reduce AST, ALT, and APN and remit the pathological changes of liver tissue [20]. In our study, it was observed that ECD can significantly reduce the body weight and abdominal circumference in mice fed with high-fat diet, improve glucose tolerance, promote the secretion of insulin and reduce the level of blood lipid, and also promote the expression of CDKAL1 mRNA and protein.

MS refers to the clinical syndrome gathered by abnormal glucose metabolism, hypertension, and lipid metabolism disorder, which takes the insulin resistance as the basis [21] and central obesity as the main performance [22]. When the insulin secretion decreased and the blood glucose increased, the apoB100 saccharification happened and then resulted in the decline of the LDL clearance by apoB receptor-mediated in liver cells and the increase in LDL-c. While the MS is in the presence of insulin resistance, the liver synthesis, and secretion of VLDL, TG is increasing, and the clearance decreases, so as to produce hyperlipidemia. It is found in this study that ECD can significantly decrease the body weight and abdominal circumference in mice fed with high-fat diet, and it is surmised that it reduces the weight and improves abdominal obesity by reducing abdominal fat accumulation. At the same time in this study, ECD improves the OGTT and decreases the TG and TC. So it is surmised that ECD can reduce blood glucose through the promotion of insulin secretion and result in the improvement of the apoB saccharification, so as to improve the insulin resistance and reduce the secretion of VLDL and TG in liver, ultimately improving hyperlipidemia.

It was reported that the ms2t6A modification of tRNALys (UUU) by CDKAL1 is required for the accurate translation of AAA and AAG codons. The human insulin gene contains 2 Lys (AAG) codons. One of the Lys residues is located at the cleavage site between the C-peptide and A chain of insulin. And it was confirmed that the defects of CDKAL1 in *β*-cell easily misread this Lys codon by ms2t6A modification-deficient tRNALys (UUU) which results in the misfolding or miscleavage of proinsulin and leads to decrease in insulin secretion and impairment of glucose regulation eventually [13, 23]. It has been reported that CDKAL1 risk allele carriers display an insulin secretory defect that is concomitant with higher levels of proinsulin [24], while some studies have shown that CDKAL1 can also promote the generation of ATP and control the insulin release within the first phase of pancreatic *β*-cells [25, 26], which has also been confirmed in the study of gene knockout mice [13]. The first phase insulin secretion is important to reduce the postprandial blood glucose, which can inhibit the production of endogenous glucose and inhibit the increase of postprandial blood glucose levels, and participate in the occurrence of high blood glucose after meal. Another animal study suggested that CDKAL1 gene deletion is accompanied by modestly impaired insulin secretion and longitudinal fluctuations in insulin sensitivity during high-fat feeding in mice. CDKAL1 may affect such compensatory mechanisms regulating glucose homeostasis through interaction with diet [27]. Additionally, several genome-wide association analyses identified that CDKAL1 multiple single nucleotide polymorphism loci were associated with human susceptibility of type 2 diabetes [28–32]. Thus far, several human studies have indicated that the risk variant of CDKAL1 is associated with reduced insulin secretion [26, 33–36].

In this study, the expressions of CDKAL1 mRNA and protein in the liver visceral adipose and subcutaneous adipose tissues of mice were significantly downregulated after being induced by high-fat diet. And after the treatment of ECD, the expressions of CDKAL1 mRNA and protein in the liver visceral adipose and subcutaneous adipose tissues of mice increased significantly. At the same time, it is found in this study that the insulin secretion in mice is reduced after inducement by the high-fat diet, and ECD can promote mouse insulin secretion. These observations suggested that ECD can increase the expression of CDKAL1 to promote the secretion of insulin, which can improve glucose metabolism.

There are several limitations in the present study. Above all, although ECD can promote the expression of CDKAL1, the mechanism of how ECD triggered the increase of CDKAL1 was still unclear. Also why ECD can regulate lipid metabolism and how ECD can adjust lipid metabolism have not been investigated in our study.

In summary, ECD has good therapeutic effects on the metabolic disorders induced by high-fat diet in mice, including reducing body weight and abdominal circumference, improving glucose tolerance, and regulating glucose and lipid metabolism. At the same time, it was observed to improve the function of islet cell by regulating the expression of CDKAL1, so as to promote the secretion of insulin. This research preliminarily studied the potential mechanism of ECD from the blood biochemical levels and the expression of CDKAL1, and further research on other related mechanisms and related pathways should be taken to provide a more effective basis for the clinical application of ECD.

## Figures and Tables

**Table 1:** List of primers.

Gene	Forward primer	Reverse primer
CDKAL1	ATCGGGGTTCAGCAGATAGAT	TCTTCGGCAAATCCAGTCGAG
*β*-actin	GGCTGTATTCCCCTCCATCG	CCAGTTGGTAACAATGCCATGT

## Abbreviations

ALT: Almandine aminotransferase
ApoB100: Apolipoprotein B100
APN: Aminopeptidase
AST: Aspartate aminotransferase
CDKAL1: CDK5 Regulatory Subunit Associated Protein 1 Like 1
HDL-C: High-density lipoprotein cholesterol
LDL-C: Low-density lipoprotein cholesterol
MS: Metabolic syndrome
OGTT: Oral glucose tolerance test
TC: Total cholesterol
TCM: Traditional Chinese Medicine
TG: Triglyceride
VLDL: Very low-density lipoprotein.
